# Understanding the Preclinical Efficacy of Antibody–Drug Conjugates

**DOI:** 10.3390/ijms252312875

**Published:** 2024-11-29

**Authors:** Cristina Díaz-Tejeiro, Alfonso López de Sá, Elisa Poyatos-Racionero, Pablo Ballestín, Jorge Bartolomé, Emiliano Calvo, Víctor Moreno, Francisco Moris, Pedro Pérez-Segura, Balazs Gyorffy, Atanasio Pandiella, Alberto Ocaña

**Affiliations:** 1 Experimental Therapeutics Unit, Oncology Department, Hospital Clínico San Carlos (HCSC), Instituto de Investigación Sanitaria San Carlos (IdISSC), 28040 Madrid, Spain; 2 Medical Oncology Department, Hospital Clínico San Carlos (HCSC), Instituto de Investigación Sanitaria San Carlos (IdISSC), 28040 Madrid, Spain; 3 Cancerappy S.L., 48950 Erandio, Spain; 4 START Madrid Centro Integral Oncológico Clara Campal, 28050 Madrid, Spain; 5 START Madrid-FJD, Hospital Fundación Jiménez Díaz, 28040 Madrid, Spain; 6 Department of Biophysics, Medical School, University of Pecs, H-7624 Pecs, Hungary; 7 Department of Bioinformatics, Semmelweis University, H-1094 Budapest, Hungary; 8 Research Centre for Natural Sciences, Cancer Biomarker Research Group, Institute of Enzymology, Magyar Tudósok Krt. 2, H-1117 Budapest, Hungary; 9 Cancer Research Center, CSIC-University of Salamanca, 37007 Salamanca, Spain; 10 Centro de Investigación Biomédica en Red en Oncología (CIBERONC), 28029 Madrid, Spain

**Keywords:** antibody–drug conjugates, tumor-associated antigens, payload, cancer, new therapies

## Abstract

Antibody–drug conjugates (ADCs) represent a therapeutic modality that guides chemotherapies to tumoral cells by using antibodies against tumor-associated antigens (TAAs). The antibody and the chemotherapy or payload are attached by a chemical structure called the linker. The strategy for the development of this type of drug was based on several rational pillars, including the use of a very potent payload and the use of specific antibodies acting only on antigens expressed on tumoral cells. In this article, by using data from all approved ADCs that have received regulatory approval, we analyze the potential contribution of each ADC component to preclinical activity. We suggest that payload potency and the drug-to-antibody ratio (DAR) have a less relevant role in relation to efficacy than previously considered. Additionally, we have observed that some ADCs have been developed against antigens also present in non-transformed tissues, which could suggest that TAA specificity is not a mandatory requirement. Finally, we have identified that ADCs with payloads harboring more favorable physicochemical characteristics showed better potential activity. In this article, we also review other aspects that should be taken into consideration for ADC design, including linker structure, stability, conjugation type, pharmacokinetics, receptor internalization, and recycling. Based on currently available data, our study summaries different concepts that should be considered in the design of novel ADCs in the future.

## 1. Introduction

Chemotherapy is still the standard of care (SOC) treatment for many solid and hematological malignancies [1,2]. This family of agents act mainly on biological functions related to cell division and DNA integrity, taking advantage of the fact that tumoral cells have higher proliferation rates compared to non-transformed ones [1,2]. Of note, although chemotherapy has been demonstrated to be highly active in many cancers, the main limitation of this type of agent is associated with the narrow therapeutic index produced by the activity of these compounds on non-tumoral tissues [3]. In this context, tissues with high cell turnover are those mainly affected by chemotherapy, including the bone marrow or epithelial tissues, leading to the presence of side effects like neutropenia, thrombopenia, or mucositis [4]. To avoid this limitation, options to improve the delivery of the chemotherapy to reach specific tumor tissues were implemented, including modifications in the schedule of administration or the infusion rate or the development of novel formulations [2,4]. These approaches have been demonstrated to be useful in some circumstances, as is the case for liposomal formulations of doxorubicin that reduce the cardiac toxicity observed with the free compound [5].

In the late 1980s, the idea of using antibodies to specifically guide chemotherapies with very high potency to specific tumor tissues was implemented [6]. These novel treatment moieties were termed antibody–drug conjugates (ADCs). ADCs consist of an antibody specifically designed against a tumoral protein, termed tumor-associated antigen (TAA), expressed at the cellular surface and a potent chemotherapeutic payload that is bound through a chemical entity called a linker [7]. This approach was based on some preliminary assumptions: (i) The higher the amount and potency of the payload, the better, as more toxic chemotherapy will reach the tumor, augmenting the therapeutic index, and (ii) the more selective the TAA compared to normal tissue, the better, to avoid on target off tumor toxicities of the antibody acting on non-tumoral cells.

With these two assumptions considered as the gold standard, most of the research performed in this field during the last twenty years has focused on identifying ways to improve them, including the identification of surface tumor proteins to design specific antibodies, the development of strategies to augment the payload by increasing the drug-to-antibody ratio (DAR), or by improving the release of the chemotherapeutic payload by using cleavable linkers.

In this article, we review how the ADC components can influence their mechanism of action and, subsequently, their potential activity through a detailed evaluation of the available data from approved ADCs. Here, we propose options for improvement and describe unknown areas that required further evaluation.

## 2. Results

### 2.1. Selective Expression of TAAs for Approved ADCs

**Figure 1:**
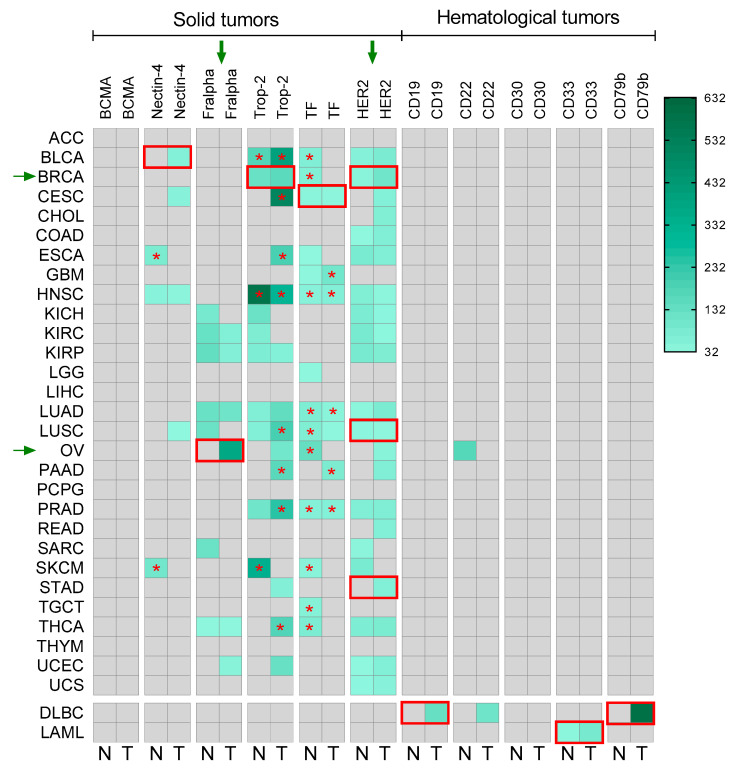
Heatmap representation of the ADC target expression approved in normal (N) and tumoral (T) tissue. Bordered in red the target expression with the indication where it is approved. The “*” shows higher expression in that tissue than in the indication approved. Green arrows indicate ADCs that were approved in patients with a specific cut-off selection by the target.

One of the classical concepts for the development of ADCs considers that the expression of the antigen is ideally exclusive from tumor cells or at least it should be overexpressed with respect to normal cells, to avoid off-target toxicities. Using transcriptomic data from public repositories, we evaluated the expression of the target from available ADCs in tumors and normal tissue. As can be seen in Figure 1, for some ADCs that have reached the clinical setting, there is no exclusive expression of the TAA in tumors. For instance, Sacituzumab govitecan against Trop2 was approved in breast cancer (BRCA), but also high expression levels of the TAA can be found in non-transformed tissue like head and neck epithelium (HNSC-normal) (Figure 1). In a similar way, for agents like Enfortumab vedotin, targeting NECTIN-4, which is approved in bladder cancer (BLCA), high expression of this TAA is also observed in the esophagus epithelium (CESC-normal). Similar findings can be observed for ADCs targeting tissue factor (TF) that is highly expressed in normal tissue (Figure 1). As discussed elsewhere [8], in other tumor types beyond those where the ADC has been approved, higher expression of the TAA is also identified, like in BLCA, CESC, HNSCC, LUSC, PAAD, PRAD, and THCA for Trop2 and ESCA and SKCM for Nectin-4 (Figure 1). TAA in hematological malignancies showed a very specific presence in line with the limited clonal heterogeneity of these malignancies (Figure 1).

### 2.2. Payload Potency and Drug-to-Antibody Ratio (DAR) in Relation to Preclinical Activity

**Figure 2:**
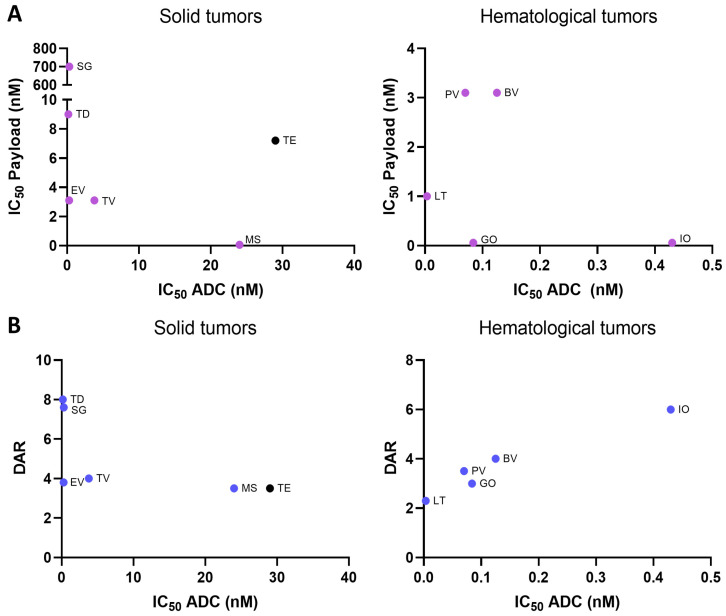
(**A**) Payload potency (IC_50_) related to preclinical activity (IC_50_ ADCs) divided for ADCs for solid (Rho: −0.3448; *p*-value: 0.5033) and hematological (Rho: −0.3936; *p*-value: 0.5121) indications, and (**B**) drug–antibody ratio (DAR) in relation to preclinical activity (IC_50_ ADCs) divided for ADCs for solid (Rho: −0.6104; *p*-value: 0.1981) and hematological indications (Rho: 0.9000; *p*-value: 0.0833). Abbreviations: Brentuximab vedotin (BV); Enfortumab vedotin (EV); Gemtuzumab ozogamicin (GO); Inotuzumab ozogamicin (IO); Loncastuximab tesirine (LT); Mirvetuximab soravtansine (MS); Polatuzumab vedotin (PV); Sacituzumab govitecan (SG); Tisotumab vedotin (TV); Trastuzumab deruxtecan (TD); Trastuzumab emtansine (TE). Black dots represent cleavable linkers of the ADC. Rho and Spearman’s *p*-value data are presented.

We next explored whether there was a correlation between in vitro potency of the payload and the ADC. All ADC features are shown in Table S1, Supplementary Materials. We first observed that there was no clear correlation between the in vitro potency of the payload and the ADC, neither in solid tumors nor hematological malignancies (Rho: −0.3448; *p*-value: 0.5033 and Rho: −0.3936; *p*-value: 0.5121, respectively) (Figure 2A). Some ADCs like Enfortumab vedotin, Loncastuximab tesirine, or Gemtuzumab ozogamicin had a potent payload and a high ADC activity. However, others like Sacituzumab govitecan, Trastuzumab deruxtecan, Polatuzumab vedotin, or Brentuximab vedotin had a potent ADC activity with a low payload killing (Figure 2A). Next, we decided to explore if ADCs with higher DAR were those with more potent ADC in vitro activity. As can be seen in Figure 2B, no relationship existed between DAR and in vitro ADC IC_50_ (solid tumors: Rho: −0.6104; *p*-value: 0.1981). Compounds with low DAR had a profound potency, like Loncastuximab tesirine, Gemtuzumab ozogamicin, or Polatuzumab vedotine. Of note, only in hematological malignancies was there a trend for a positive association (Rho: 0.9000; *p*-value: 0.0833). These data clearly demonstrate that, for the in vitro killing capacity, several other factors must be taken into consideration, including receptor internalization or the receptor recycling rate, areas that have not been fully evaluated.

### 2.3. Payload Potency and Drug-to-Antibody Ratio (DAR) in Relation to Clinical Activity

**Figure 3:**
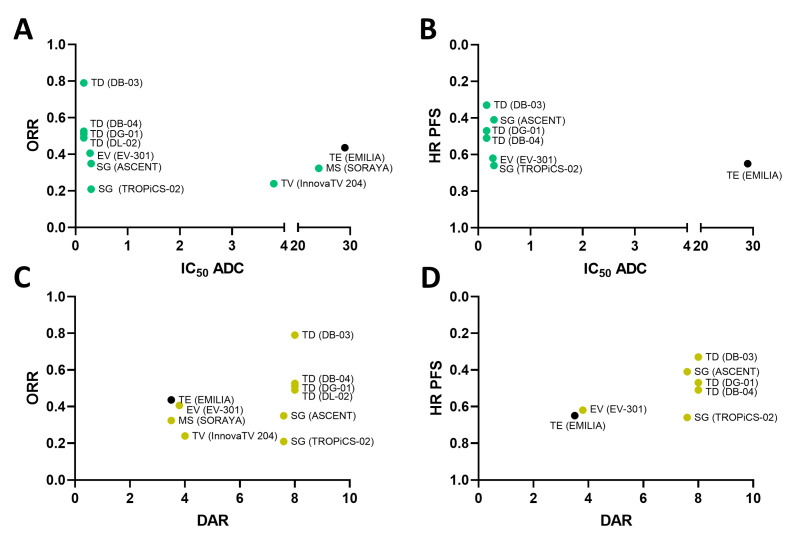
(**A**) Relation between ORR and IC_50_ ADC (Rho: −0.1803; *p*-value: 0.6181) and (**B**) HR PFS (Rho: 0.4481; *p*-value: 0.3132) with IC_50_ ADC. (**C**) Relation between ORR (Rho: 0.4299; *p*-value: 0.2149) and DAR; (**D**) Relation between HR PFS (Rho: −0.6394; *p*-value: 0.1221) and DAR. Abbreviations: Enfortumab vedotin (EV); Mirvetuximab soravtansine (MS); Sacituzumab govitecan (SG); Tisotumab vedotin (TV); Trastuzumab deruxtecan (TD); Trastuzumab emtansine (TE). Black dots represent cleavable linkers of the ADC. In parentheses are the clinical trial. Rho and Spearman’s *p*-value data are presented.

In line with this, we explored if the ADC potency was associated with the clinical activity observed in patients, including endpoints used for their approval, either PFS or ORR (for those approved with an accelerated path, or ORR reported in phase III clinical studies). No association was observed between ADC potency and clinical benefits (Figure 3A) (ORR: Rho: −0.1803; *p*-value: 0.6181 and HR for PFS: Rho: 0.4481; *p*-value: 0.3132). Most of the ADCs, with the exception of Trastuzumab emtansine, displayed high potency in vitro but very different levels of clinical activity.

One of the strategies to improve the efficacy of ADCs includes the increase in the amount of the payload by augmenting the DAR. In this context, some ADCs were designed to have a high DAR, with the intention to increase the clinical efficacy. In our analysis displayed in Figure 3B, agents with high DAR displayed a wide range of clinical activity, without clear correlation between DAR and clinical activity (ORR: Rho: 0.4299; *p*-value: 0.2149 and HR for PFS: Rho: −0.6394; *p*-value: 0.1221).

### 2.4. Dose Selection of Approved ADC

**Figure 4:**
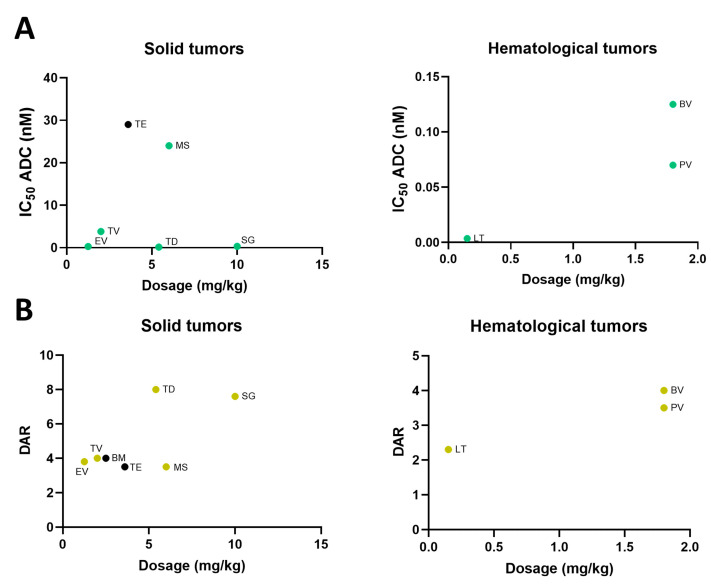
(**A**) Pearson correlation of clinical doses with ADC potency divided into ADCs for treatment for solid (Rho: −0.05069; *p*-value: 0.9240) and hematological indications (Rho: 0.891; *p*-value: 0.2989) and (**B**) Pearson correlation of clinical doses with DAR divided into ADCs for treatment for solid (Rho: 0.6785; *p*-value: 0.0938) and hematological indications (Rho: 0.9582; *p*-value: 0.1847). Abbreviations: Belantamab mafodotin (BM); Brentuximab vedotin (BV); Enfortumab vedotin (EV); Loncastuximab tesirine (LT); Mirvetuximab soravtansine (MS); Polatuzumab vedotin (PV); Sacituzumab govitecan (SG); Tisotumab vedotin (TV); Trastuzumab deruxtecan (TD); Trastuzumab emtansine (TE). Black dots represent cleavable linkers of the ADC. Rho and Spearman’s *p*-value data are presented.

We next explored the relationship between in vitro ADC potency, or the DAR, and the human doses reached in clinical studies. It would be expected that a high ADC potency or DAR could be associated with a lower dose needed in studies that were used for the approval of the ADC. As can be seen in Figure 4A, some ADCs in solid and hematologic malignancies displayed a high ADC potency with high administered doses in patients, so no correlation was clearly observed (Rho: −0.05069; *p*-value: 0.9240 in solid tumors and Rho: 0.8918; *p*-value: 0.2989 for hematology malignancies). As an example, Sacituzumab govitecan showed a high potency in vitro but needed high doses in patients, or trastuzumab emtansine displayed a low ADC potency and, in contrast, needed low doses of the compound in patients. Similar findings were observed in hematological malignancies, as is the case for Brentuximab vedotin and Polatuzumab vedotin (Figure 4A).

In line with the previous analysis, we explored the relationship between DAR and approved doses. In a similar way, we did not observe any association (Rho: 0.6785; *p*-value: 0.0938 in solid tumors and Rho: 0.9582; *p*-value: 0.1847 in hematological malignancies). ADCs with high DAR needed high doses like Sacituzumab govitecan, or, by contrast, ADCs with low DAR needed low doses like Loncastuximab tesirine (Figure 4B).

### 2.5. Evaluation of ADCs with Adequate Physicochemical Properties and Conjugation Type

In a previous study, we evaluated which approved ADCs contained payloads with favorable physicochemical properties (PCPs) [9]. Payloads with more favorable PCPs were considered those that did not violate any parameter of the Lipinski rules [10]. For a detailed definition of PCP, we refer to the Materials and Methods section. Only two ADCs, Trastuzumab deruxtecan and Sacituzumab govitecan, demonstrated adequate properties, considering also the Ghose, Veber, Egan, and Muegge rules [9]. In addition, we also studied the conjugation type, including stochastic conjugation or homogeneous conjugation (HC). All ADCs had stochastic conjugation, except for Trastuzumab deruxtecan and Sacituzumab govitecan. These two ADCs were also those with good PCP.

**Figure 5:**
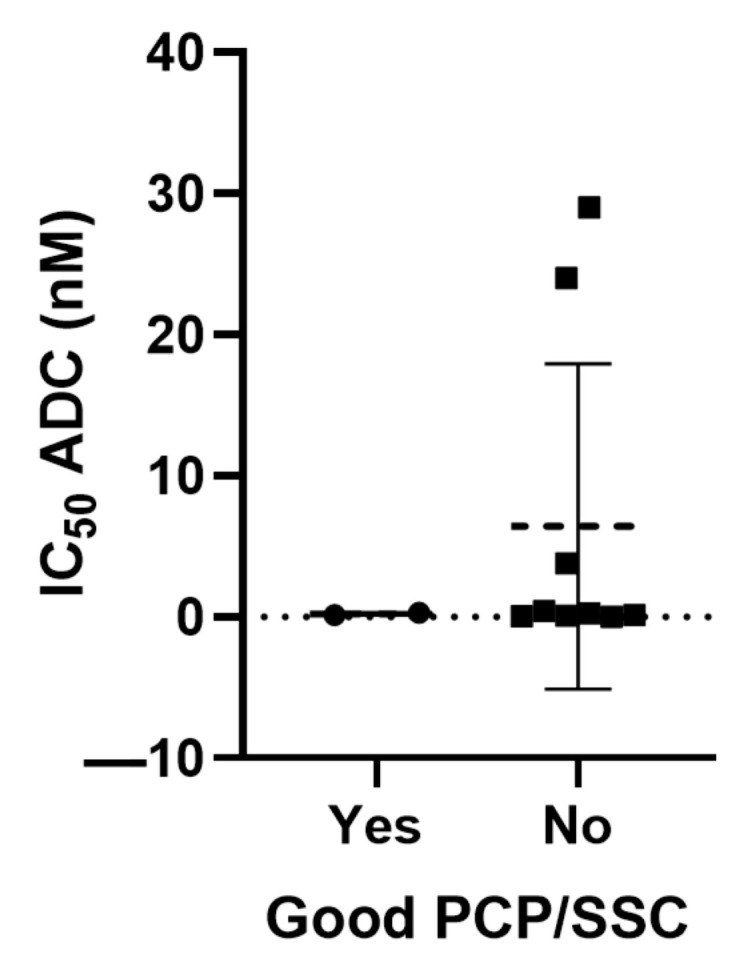
Good PCP/SSC with IC_50_ ADC (nM). Unpaired *t*-tests were performed to analyze differences between groups.

We then compared the in vitro ADC killing potency of Trastuzumab deruxtecan and Sacituzumab govitecan with the rest of the ADCs. As can be seen in Figure 5, these two ADCs displayed high potency.

## 3. Discussion

In the present article, we have analyzed the potential contribution to the preclinical activity of different ADC components, with the aim of gaining insights about how to improve the development of this kind of agent.

We first observed that TAAs used as targets for approved ADCs did not need to be specific or overexpressed in tumoral areas to reach clinical activity in patients. For instance, NECTIN-4, the TAA of Enfortumab-vedotin that is approved for bladder cancer, displayed higher expression in some non-transformed tissues like esophagus epithelium (CESC-normal) than in the tumor; or Trop2 the TAA of Sacituzumab-govitecan that is approved in breast cancer (BRCA), also showed high expression levels in head and neck epithelium (HNSC-normal). Other examples are provided in Figure 1. These findings challenge the general concept that recommends selecting only specific TAA for the design of novel ADCs to avoid on-target off-tumor toxicities.

We next explored if the payload potency or DAR was associated with ADC potency. It has been considered that those ADCs with the highest payload potency and with the highest DAR could have a greater in vitro killing. In our evaluation, we did not observe this association, neither in solid nor in hematologic malignancies.

In our subsequent analysis, we explored whether the physicochemical properties of the ADC payloads could influence the ADC in vitro efficacy. In a previous study, we identified which ADCs had payloads with favorable PCPs following the standard chemical rules [9]. Only two ADCs harbored payloads with more favorable physicochemical properties, Trastuzumab deruxtecan and Sacituzumab govitecan [9]. In this article, we show that those ADCs clearly have a more potent ADC killing activity. Of note, both ADCs have cleavable linkers and homogeneous conjugation, so their payloads can much more easily diffuse through intracellular and extracellular membranes acting on cells that do not express the TAA.

Globally, our analysis reveals that there are concepts regarding the mechanism of action of ADCs that have not been taken into consideration as deserved. For instance, the internalization rate, recycling rate, and subcellular trafficking could be more important in relation to clinical activity than previously thought. In this regard, recent articles suggest the importance of these biological processes that can vary among receptors and also tumoral types [11]. In this context, an exclusive expression of the target or a high DAR would not be as relevant as has been classically considered. Some articles have recently reviewed the impact of endocytosis in the mechanism of action of ADCs, and some researchers, including our group, have described their implication in the mechanism of resistance of some ADCs [11,12,13]. Differences in these processes could undoubtedly impact the presence of a free payload in tumoral areas and therefore justify the differences observed in our analysis in the in vitro killing. Recent attention has been given to other forms of delivery, including antibody nanoconjugates, as this approach could reduce some of these limitations [14,15]. Our group has been pioneered in the design of this kind of agents [16]. Potential benefits include the fact that no endocytosis processes would be required as nanoparticles will passively diffuse through the plasma membrane [17]. A better understanding of these mechanisms could not only permit the design of optimized ADCs to improve endocytosis but would also help in the selection of the population based on the expression levels of the target. In line with this target, heterogeneity and drug penetration must be taken as well into consideration [18]. Only two ADCs have been approved in indications where patients were selected by a particular TAA cut-off expression level (Figure 1 and Supplementary Table S1). Those included Mirvetuximab soravtansine and trastuzumab emtansine and deruxtecan against TF and HER2, respectively [19,20,21]. The presence of payloads with adequate physicochemical characteristics and cleavable homogeneous linkers could facilitate the selection of patients without a particular cut-off level of the target if internalization of the receptor and endocytosis rate is adequate. Of note, recently, trastuzumab deruxtecan has demonstrated activity in ultra-low HER2 tumors in first-line advanced breast cancer (DESTINY-Breast06) [22].

Artificial intelligence could help in different steps of the process of ADC design. These can include the integration and analysis of preclinical data like preclinical efficacy data (IC_50_ values), PK/PD data, and preclinical toxicology information. AI can also help in the simulations of ADC interactions and in the optimization of ADC design by predicting the best combination of antibody, payload, and linkers.

Our study has limitations. For instance, an evaluation of the pharmacokinetic properties of each ADC in preclinical and clinical models could help understand the differences observed. Genomic data, although, in most occasions, correlate with a protein expression, are not the best way to evaluate target presence. Additionally, there is a lack of published data to assess key factors influencing ADC activity, such as internalization and endocytosis rates or the stability of linkers. Of note, the data published and incorporated in this analyses, has been provided by sponsors to regulatory agencies with the potential for heterogeneity that could be present Of note, very recently relevant concepts regarding the therapeutic index of ADC have been described, suggesting that ADCs do not improve the therapeutic index compared to payloads, a concept that has implications for safety as this is the main limitation for the development of these agents [23]. Lastly, we recognize that toxicity, one of the main limiting factors in ADC development, has not been assessed in this article [24].

In conclusion, we have highlighted key factors that can impact ADC effectiveness and proposed areas for further research that may inform future ADC design and optimization.

## 4. Materials and Methods

### 4.1. Data Extraction

In order to identify FDA-approved ADC, we researched the FDA website (last access June 2024). The FDA has a publicly available database termed “Novel Drug Approvals” that is updated every year. We thoroughly examined the available list to identify drugs that are considered ADC and have been approved.

### 4.2. Transcriptomic Extraction and Data Analysis

We selected those targets which TPM (transcripts per million) were equal to or greater than 32, a value considered medium/high in terms of gene expression experiments (PCR). We used the GEPIA dataset (Gene Expression Profiling Interactive Analysis; http://gepia2.cancer-pku.cn/ using TCGA, last accessed on 1 April 2024) to analyze the expression (TPM) of these targets in the tumoral and non-transformed (normal) tissue of all cancer types [25].

### 4.3. Extraction, Collection, and Analysis of Preclinical and Clinical Data from ADCs

We conducted a search on the FDA website (https://www.fda.gov/drugs/drug-approvals-and-databases/resources-information-approved-drugs) on 1 April 2024 to identify FDA-approved ADCs from recent years. For each ADC identified, we used PubMed (https://pubmed.ncbi.nlm.nih.gov) to locate the clinical trials that supported its regulatory approval. Our preclinical data analysis was limited to in vitro cytotoxicity assays, as reported by the sponsors and available within the FDA label documentation.

### 4.4. Evaluation of Physicochemical Properties

All ADME parameters (Absorption, Distribution, Metabolism, and Excretion), including the Lipinski rule, were calculated using SwissADME, a free software program available from the Swiss Institute of Bioinformatics [26]. In addition, each of the parameters that constitute the Lipinski rule were broken down. Also, the average calculation of the LogP and LogS parameters obtained by different algorithms in the software was included, to highlight the lipophilicity and solubility of each payload. Given that certain ADCs have cleavable linkers, others have non-cleavable ones, and some can be developed with either type, the molecular structure of the released payload has been considered in the calculation of the ADMET parameters. This includes the linker fragment and/or residual amino acids that remain once the payload is released. Moreover, when the corresponding payloads are available in both cleavable and non-cleavable forms, both forms are included in the calculations.

### 4.5. Statistical Analysis

All data were analyzed using the statistical software GraphPad Prism 7 with the unpaired *t*-test for independent samples and the correlations analysis by Spearman. Rho and *p*-values are shown. Unpaired *t*-tests were performed to analyze differences between groups. The level of significance was considered 95% (* *p* ≤ 0.05).

## Data Availability

All data generated or analyzed during this study are included in this published article.

## Supplementary Materials

The following supporting information can be downloaded at https://www.mdpi.com/article/10.3390/ijms252312875/s1.

## Abbreviations

ACC	Adrenocortical carcinoma
ADCs	Antibody–drug conjugates
ADME	Absorption, Distribution, Metabolism, and Excretion
BLCA	Bladder Urothelial Carcinoma
BRCA	Breast invasive carcinoma
BV	Brentuximab vedotin
CESC	Cervical squamous cell carcinoma and endocervical adenocarcinoma
CHOL	Cholangiocarcinoma
COAD	Colon adenocarcinoma
DAR	drug antibody ratio
DLBC	Lymphoid Neoplasm Diffuse Large B-cell Lymphoma
ESCA	Esophageal carcinoma
EV	Enfortumab vedotin
GBM	Glioblastoma multiforme
GO	Gemtuzumab ozogamicin
HNSC	Head and Neck squamous cell carcinoma
IO	Inotuzumab ozogamicin
KICH	Kidney Chromophobe
KIRC	Kidney renal clear cell carcinoma
KIRP	Kidney renal papillary cell carcinoma
LAML	Acute Myeloid Leukemia
LGG	Brain Lower Grade Glioma
LIHC	Liver hepatocellular carcinoma
LT	Loncastuximab tesirine
LUAD	Lung adenocarcinoma
LUSC	Lung squamous cell carcinoma
MS	Mirvetuximab soravtansine
OV	Ovarian serous cystadenocarcinoma
PAAD	Pancreatic adenocarcinoma
PCP	Physicochemical properties
PCPG	Pheochromocytoma and Paraganglioma
PRAD	Prostate adenocarcinoma
PV	Polatuzumab vedotin
READ	Rectum adenocarcinoma
SARC	Sarcoma
SG	Sacituzumab govitecan
SKCM	Skin Cutaneous Melanoma
SOC	standard of care
STAD	Stomach adenocarcinoma
TAAs	Tumor-associated antigens
TD	Trastuzumab deruxtecan
TE	Trastuzumab emtansine
TF	tissue factor
TGCT	Testicular Germ Cell Tumors
THCA	Thyroid carcinoma
THYM	Thymoma
TPM	transcripts per million
TV	Tisotumab vedotin
UCEC	Uterine Corpus Endometrial Carcinoma
UCS	Uterine Carcinosarcoma
