# Supplementary figures and images for: Understanding the Preclinical Efficacy of Antibody–Drug Conjugates
Source: Int J Mol Sci. 2024 Nov 29;25(23):12875. doi: 10.3390/ijms252312875 (PMC11641021; doi:10.3390/ijms252312875)

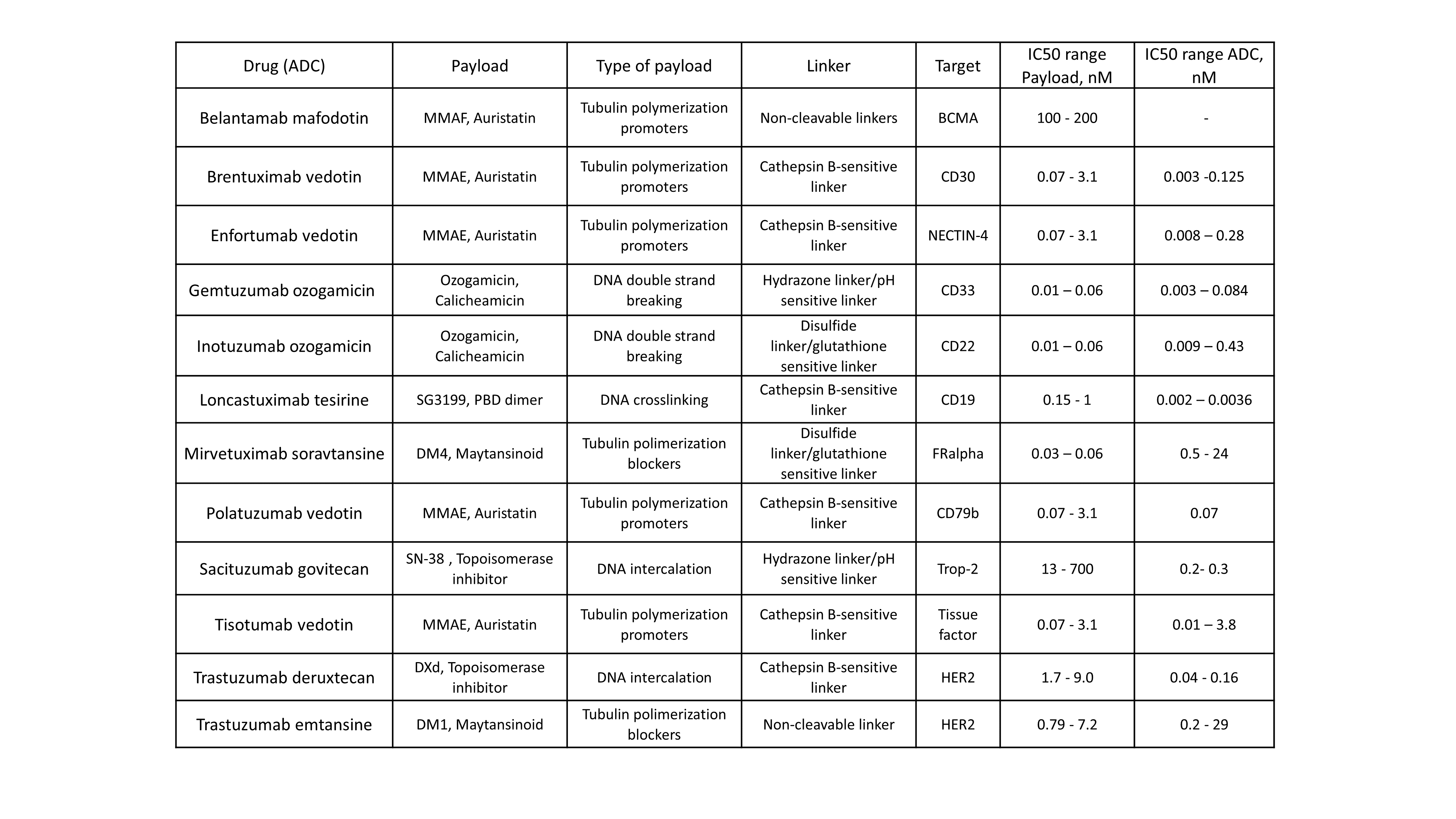

Supplement: Supplementary file 1 [file ijms-25-12875-s001.zip › ijms-3339081-supplementary.png]
